# Genome-wide profiling of patient-derived glioblastoma stem-like cells reveals recurrent genetic and transcriptomic signatures associated with brain tumors

**DOI:** 10.1007/s11060-023-04287-6

**Published:** 2023-05-04

**Authors:** Elisabetta Lazzarini, Domenico Alessandro Silvestris, Giuseppe Benvenuto, Daniela Osti, Luigi Fattore, Rosina Paterra, Gaetano Finocchiaro, Paolo Malatesta, Antonio Daga, Alberto L. Gallotti, Rossella Galli, Giuliana Pelicci, Anna Tesei, Martina Bedeschi, Roberto Pallini, Lorenza Pasqualini, Chiara Romualdi, Angela Gallo, Lucia Ricci-Vitiani, Stefano Indraccolo

**Affiliations:** 1 Basic and Translational Oncology Unit, Istituto Oncologico Veneto IOV – IRCCS, via Gattamelata, 64, 35128 Padova, Italy; 2 Unit of Genetics and Epigenetic of Pediatric Cancer, Oncohaematology Department, IRCCS Ospedale Pediatrico Bambino Gesù, Viale di San Paolo 15, 00146 Rome, Italy; 3 Department of Biology, University of Padova, Padova, Italy; 4 Department of Experimental Oncology, European Institute of Oncology (IEO), IRCCS, 20139 Milan, Italy; 5 SAFU Laboratory, Department of Research, Advanced Diagnostics and Technological Innovation, Translational Research Area, IRCCS Regina Elena National Cancer Institute, Rome, Italy; 6 SC Neurologia 2- Neuroncologia- Fondazione IRCCS Istituto Neurologico Carlo Besta, Milan, Italy; 7 IRCCS Ospedale Policlinico San Martino, Genova, Italy; 8 Dipartimento di Medicina Sperimentale, Università di Genova, Genova, Italy; 9 Neural Stem Cell Biology Unit, Division of Neuroscience, IRCCS San Raffaele Hospital, Via Olgettina 58, Milan, Italy; 10 Department of Translational Medicine, University of Piemonte Orientale, Novara, Italy; 11 Biosciences Laboratory, IRCCS Istituto Romagnolo per lo Studio dei Tumori (IRST) “Dino Amadori”, Meldola, Italy; 12 Department of Neurosurgery, Fondazione Policlinico Universitario A. Gemelli IRCCS, Università Cattolica del S. Cuore, Largo A. Gemelli, 8, Rome, Italy; 13 Department of Oncology and Molecular Medicine, Istituto Superiore di Sanità, Viale Regina Elena 299, 00161 Rome, Italy; 14 Department of Surgery Oncology and Gastroenterology (DiSCOG), University of Padova, Padova, Italy

**Keywords:** Glioblastoma, GBM stem-like cells (GSCs) lines, WES, RNA-seq

## Abstract

**Purpose:**

Patient-derived cancer cell lines can be very useful to investigate genetic as well as epigenetic mechanisms of transformation and to test new drugs. In this multi-centric study, we performed genomic and transcriptomic characterization of a large set of patient-derived glioblastoma (GBM) stem-like cells (GSCs).

**Methods:**

94 (80 I surgery/14 II surgery) and 53 (42 I surgery/11 II surgery) GSCs lines underwent whole exome and trascriptome analysis, respectively.

**Results:**

Exome sequencing revealed *TP53* as the main mutated gene (41/94 samples, 44%), followed by *PTEN* (33/94, 35%), *RB1* (16/94, 17%) and *NF1* (15/94, 16%), among other genes associated to brain tumors. One GSC sample bearing a *BRAF* p.V600E mutation showed sensitivity in vitro to a BRAF inhibitor. Gene Ontology and Reactome analysis uncovered several biological processes mostly associated to gliogenesis and glial cell differentiation, S − adenosylmethionine metabolic process, mismatch repair and methylation. Comparison of I and II surgery samples disclosed a similar distribution of mutated genes, with an overrepresentation of mutations in mismatch repair, cell cycle, p53 and methylation pathways in I surgery samples, and of mutations in receptor tyrosine kinase and MAPK signaling pathways in II surgery samples. Unsupervised hierarchical clustering of RNA-seq data produced 3 clusters characterized by distinctive sets of up-regulated genes and signaling pathways.

**Conclusion:**

The availability of a large set of fully molecularly characterized GCSs represents a valuable public resource to support the advancement of precision oncology for the treatment of GBM.

**Supplementary Information:**

The online version contains supplementary material available at 10.1007/s11060-023-04287-6.

## Introduction

Glioblastoma (GBM) is a deadly malignancy which has been thoroughly characterized at the genetic level [1, 2, 3]. Patient-derived cancer cell lines can be very useful to investigate genetic as well as epigenetic mechanisms of transformation and to investigate new drugs. Previous studies have already reported the gene expression profile of gliomasphere cultures or the genetic fingerprint of GBM cell lines, but, with few exceptions [4], these were small-sized studies involving a limited (generally < 20) number of GBM cell lines [5, 6, 7]. These earlier studies had some intrinsic limitations, including (i) the fact that rare genetic alterations could have been missed, (ii) the lack of a functional characterization of the genetic alterations and (iii) the lack of paired whole-transcriptome sequencing.

In this context, the Alliance Against Cancer (ACC) Italian network promoted between 2017 and 2019 genetic characterization of a large cohort of clinically annotated patient-derived GBM stem-like cells (GSCs) available in the various laboratories of the network.

In this view, this public collection will be extremely useful for future projects to model human GBMs, and represents a valuable and accessible resource to guide the development of novel therapeutic strategies. We hereby describe the results of GSCs genomic and transcriptomic profiling, and a proof-of concept validation of a therapeutic target uncovered by genomic profiling.

## Methods

### Patients

GBM samples were obtained from 94 adult patients who underwent surgery at diagnosis (n = 80) or relapse (n = 14), from 6 centers of the ACC Italian network including Institute of Neurosurgery, Catholic University of Rome/ Istituto Superiore di Sanità, Rome; Department of Neurosurgery, Fondazione IRCCS Istituto Neurologico Carlo Besta, Milan; IRCCS Ospedale Policlinico San Martino, Genua; IRCCS Ospedale San Raffaele Scientific Institute, Milan; European Institute of Oncology, Milan and IRCCS Istituto Romagnolo per lo Studio dei Tumori “Dino Amadori”, Meldola. Constitutive DNA was obtained from blood cells in 8 GBM patients. The study on human samples was conducted in compliance with the Declaration of Helsinki and with policies approved by the Ethics Board of each hospital. Informed consent was obtained from all participants.

#### Key inclusion criteria

Diagnosis of GBM (first diagnosis or recurrence) (WHO Grade IV glioma), age 18-65, unifocal lesions, good patient’s functional status (Karnofsky performance score > 70), absence of *IDH1/IDH2* mutations, and candidate for tumor resection followed by the standard Stupp protocol.

#### Key exclusion criteria

Candidates for experimental treatments or trial, patients not eligible for surgery.

### **GBM stem-like cell cultures and** in vitro **culture conditions**

In all centers, surgical specimens were subjected to mechanical dissociation with a sterile scissor and incubated in an enzyme solution (Collagenase [Gibco], DNase1 [Roche] or papain [Worthington]) at 37 °C for up to 2 h. Tissue pieces were re-mixed by gentle pipetting at an interval of 20 min during incubation. The resulting cell suspension was cultured in serum-free DMEM/F12 medium (Dulbecco’s modified Eagle medium/ Ham’s F12 Nutrient Mixture; Gibco) containing human recombinant epidermal growth factor (hEGF) (#AF-100-15, Peprotech; 20 ng/mL), fibroblast growth factor 2 (FGF-2) (#100-18B, Peprotech; 10 ng/mL), and supplemented with B27 (Life Technologies), as previously described [8]. Cell cultures actively proliferating required 3 to 4 weeks to be established. Once established, GSCs were passaged by mechanically dissociation and grown as spheroid aggregates called neurospheres at 37 °C in a 5% CO_2_ humidified incubator.

Human GSCs from OSR were cultured in standard medium containing human recombinant EGF and FGF-2 as above, supplemented with a hormone mixture, as described in Galli et al., 2019 [9].

Actively proliferating cell cultures required 3 to 4 weeks to be established. Once established, GSC lines were subcultured by mechanical dissociation and grown as spheroid aggregates called neurospheres at 37 °C in a 5% CO_2_ humidified incubator.

### In vitro **testing of*BRAF*inhibitors**

hGBM8 (*BRAF* wild-type) and hGBM9 (p.V600E *BRAF*-mutated) GSCs were seeded at 1*10^5^ in 96-well plate in the presence of different concentrations of the BRAF inhibitor dabrafenib for 72 h. Cell viability has been determined through the CellTiter-Glo® Luminescent Cell Viability Assay (Promega). Differently, p.V600E *BRAF*-mutated A375 melanoma cells were seeded at 5*10^3^ in 96-well plate and treated with different concentrations of dabrafenib for 72 h. Quantitative analyses for curve fitting have been plotted by GraphPad Prism 7.0 (data are means ± SD).

For the neurosphere formation assay, a total of 3000 hGBM8 and hGBM9 were cultured with 5 µM of dabrafenib in methylcellulose containing medium (D-MEM/F12 medium with growth factors and an equal volume of Methylcellulose [StemCell Technologies]). Cells were incubated for 16 days at 37 °C and 5% CO_2_. Spheres were scored at the end of the incubation period, and counted using an inverted microscope and the scoring grid. Only neurospheres with a diameter > 100 μm were counted.

### Nucleic acids extraction and libraries preparation

Genomic DNA and total RNA extraction from GSCs cultures was performed with QIAamp DNA Mini kit (Qiagen) and RNeasy plus mini kit (Qiagen) respectively. Nucleic acids concentration and quality were assessed using Qubit 2.0 Fluorometer (Life Technologies) and Agilent 2100 Bioanalyzer (Agilent Technologies).

WES and RNA-seq libraries were prepared using the SureSelect Human All Exon V6 kit (Agilent Technologies) and NEBNext® Ultra™ Directional RNA Library Prep Kit (New England Biolabs) following the manufacturer’s instructions. Libraries were sequenced in paired-ends (2 × 150) on a HiSeq2500 platform (Illumina).

Methods related to WES and RNAseq data analysis are reported online as Supplementary Information.

## Results

### Genomic characterization of GBM cell lines

We characterized by WES 94 GSC cultures which were derived from adult patients undergoing surgery at diagnosis or relapse within the multi-centric study agreement supported by the ACC network. These 94 GSCs were selected from a larger cohort of GSCs (> 500) available in the various labs of the ACC network based on several criteria including (I) availability of relevant clinical information (Supplementary Table 1) and (II) tumorigenicity tested in immunodeficient mice. Altogether, we sequenced 80 GSCs obtained from primary (1st surgery, I) GBMs and 14 recurrent (2nd surgery, II) GBMs. Matched normal blood cells available from 8 patients were sequenced in order to generate a database of common genetic variants in our study population. An average of 142 932 190 reads passed the quality criteria of Q score (Phred quality score) ≥ 20. We obtained 99% of both mapped reads and properly paired reads across all cell lines and an average coverage of ~ 109X.

**Fig. 1:**
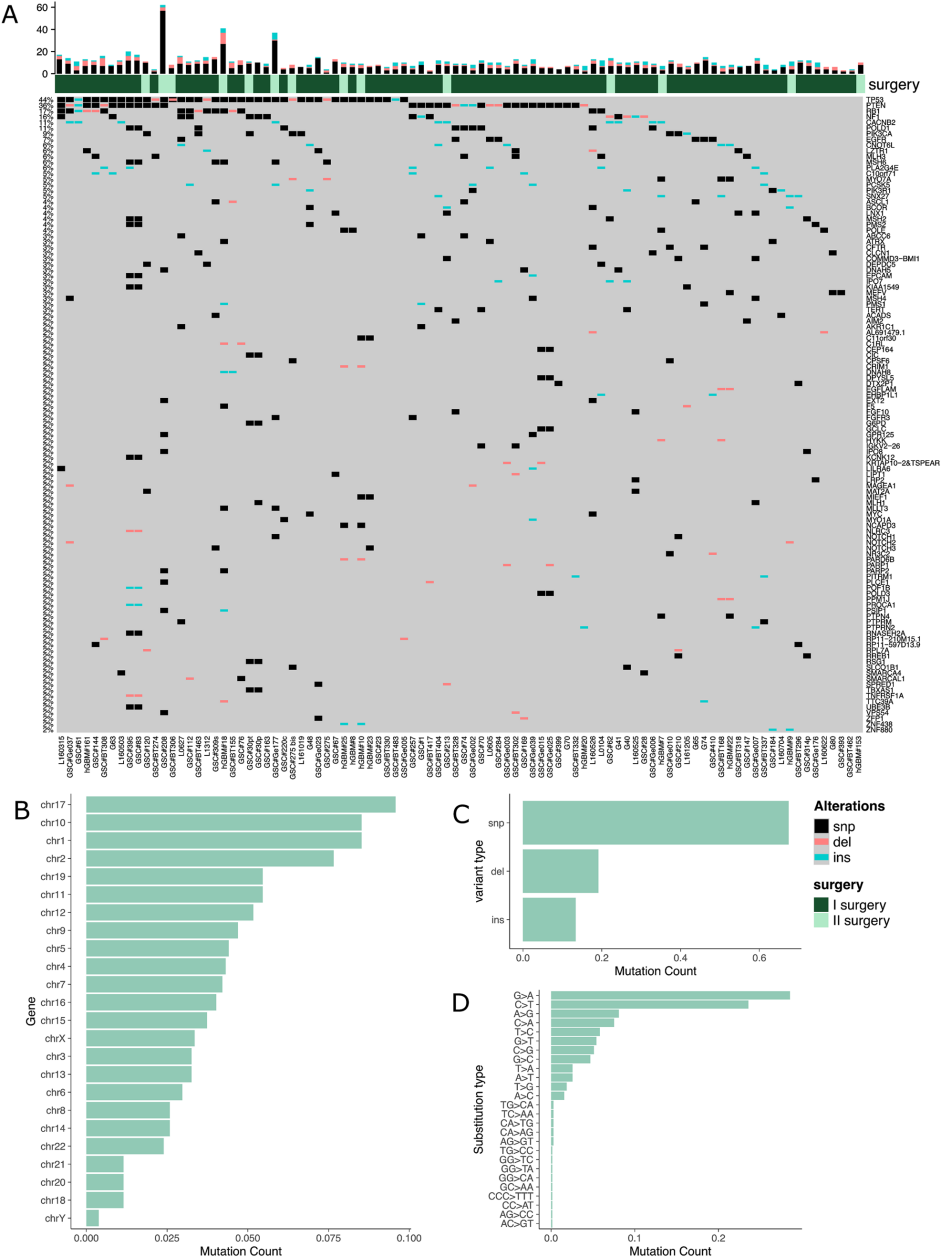
**A,** Oncoprint of the genes mutated in at least 2 samples. For each gene and for each cell line the type of mutations (snp, del or ins) is reported. On the left side part of the heatmap a bar plot of the frequency of mutation type is reported for each gene. On the upper panel a bar plot of the frequency of the mutation type is reported for each cell line. **B,** Distribution of the number of identified mutations across chromosomes. **C,** Number of mutation types identified in the entire cohort. **D,** Distribution (absolute frequencies) of the type of substitutions in the identified mutations

After the exclusion of population polymorphisms, synonymous mutations as well as non-synonymous mutations predicted to have low impact on protein functions and those variants not found in Cosmic or Varsome databases, we ended up with 889 variants in 641 genes. The top 50 mutated genes included known driver genes previously found mutated in brain tumors such as *TP53*, *PTEN*, *RB1, NF1, POLD1, PIK3CA, EGFR*, and *MSH6* among others (Fig. 1A). All chromosomes were affected by mutations, which however were predominantly found in chromosome 17 (Fig. 1B). Variants were mainly represented by single nucleotide variants (SNVs) and short insertions, and to a lesser extent by short deletions (Fig. 1C). Variants were mainly represented by G > A and C > T substitutions (Fig. 1D).

At the top of the mutated gene list, we found *TP53*, with 43 mutations in 41 GSCs (44%), followed by *PTEN* with 34 mutations in 33 GSCs (36%), *RB1* with 16 mutations in 16 GSCs (17%), and *NF1* with 15 mutations in 15 GSCs (16%) (Fig. 1A). Those genes have established roles in GBM, and show high mutation frequencies in accordance with previous studies [10, 11].

GO analysis using the complete list of mutated genes uncovered several biological processes mostly associated to gliogenesis and glial cell differentiation, including the regulation of neural precursor cell proliferation, as well as regulation of cell-substrate adhesion trough cadherin binding. Other emerging molecular processes were those regulating mismatch repair and S − adenosylmethionine metabolic process (Supplementary Fig. 1A, Supplementary Fig. 2A). Of note, S-adenosyl methionine is the principal methyl donor in cells and takes part in critical epigenetic mechanisms, including DNA methylation in the central nervous system [12].

GO molecular function and cellular component analysis uncovered alterations of DNA repair mechanisms and other less expected pathways (Supplementary Fig. 1B, C, Supplementary Fig. 2B, C). The dominant signatures of DNA repair, mismatch repair and methylation emerged also from analysis of mutated genes using Reactome and KEGG database (Supplementary Fig. 1D, E).

**Fig. 2:**
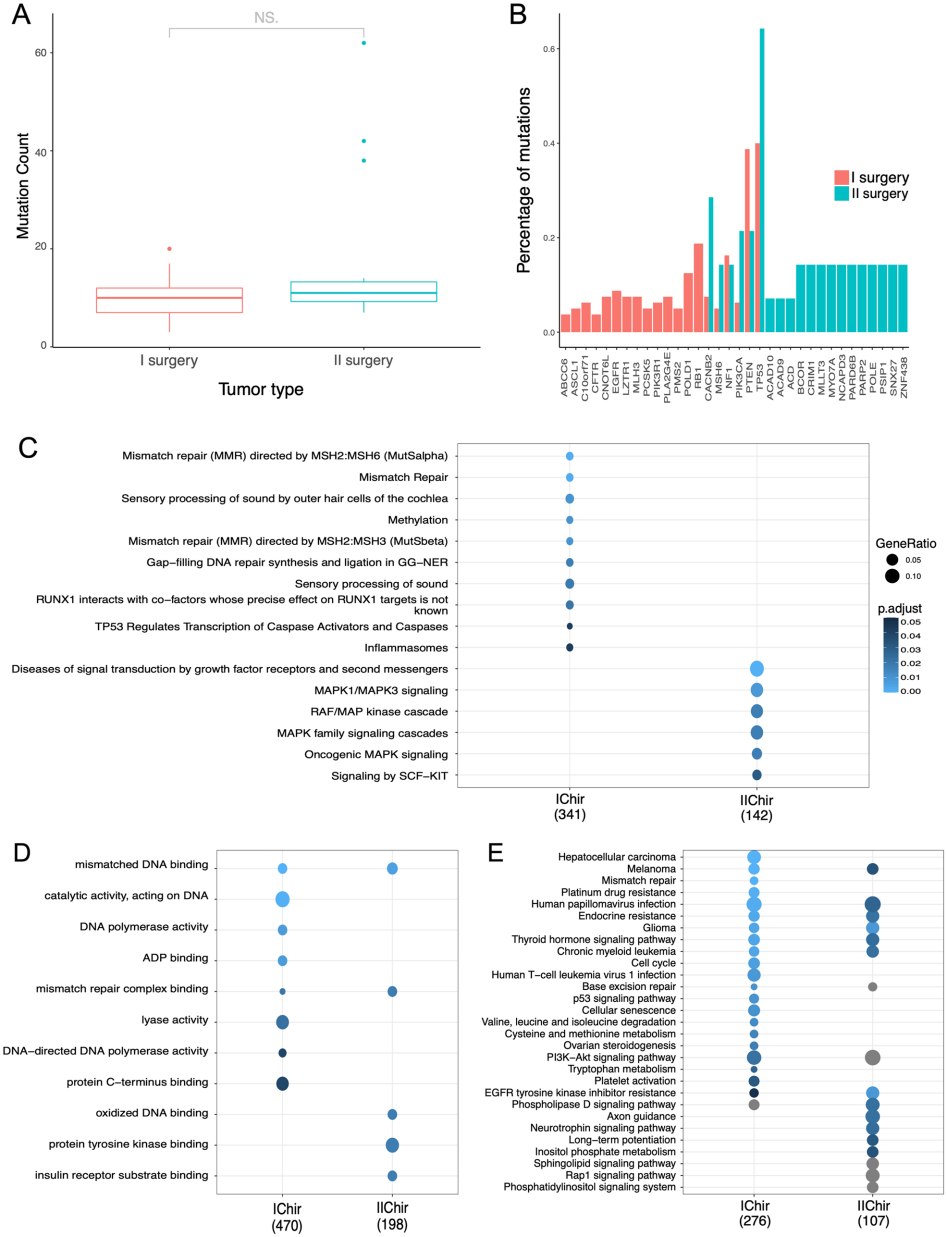
**A,** Distribution of the number of mutations per GSCs divided by sample type (I and II surgery). **B,** Bar plot of top 20 mutated genes divided by sample type (I and II surgery). **C,** Differences of GSCs derived by I and II surgery samples in terms of enriched pathways. Biological process categories **D,** Reactome pathways **E** KEGG pathways

Comparative analysis of gene ontology between GSCs from I and II surgery disclosed a similar distribution of mutated genes (Fig. 2A) but only partially overlapping top 20 mutated genes, including *NF1*, *PIK3CA*, *PTEN* and *TP53* (Fig. 2B). In three GSCs (GSC#Ge177, hGBM#18, GSC#208), all obtained at relapse from different laboratories, the number of mutations (n = 32, 40, 60, respectively) largely exceeded the average number of mutations (n = 9) found in all GSCs analyzed (Fig. 2A). Differential gene set analyses uncovered gene sets differentially represented in I and II surgery (Fig. 2C for Reactome pathways, D GO terms and E for KEGG pathways): mutations in mismatch repair, cell cycle, cellular senescence, p53 signaling pathway and methylation genes (among others) were apparently more represented in I surgery samples, whereas mutations affecting receptor tyrosine kinase signaling and ERK/MAPK signaling pathways were more represented in II surgery.

### Validation of *BRAF* mutation as an actionable marker in GSCs

**Fig. 3:**
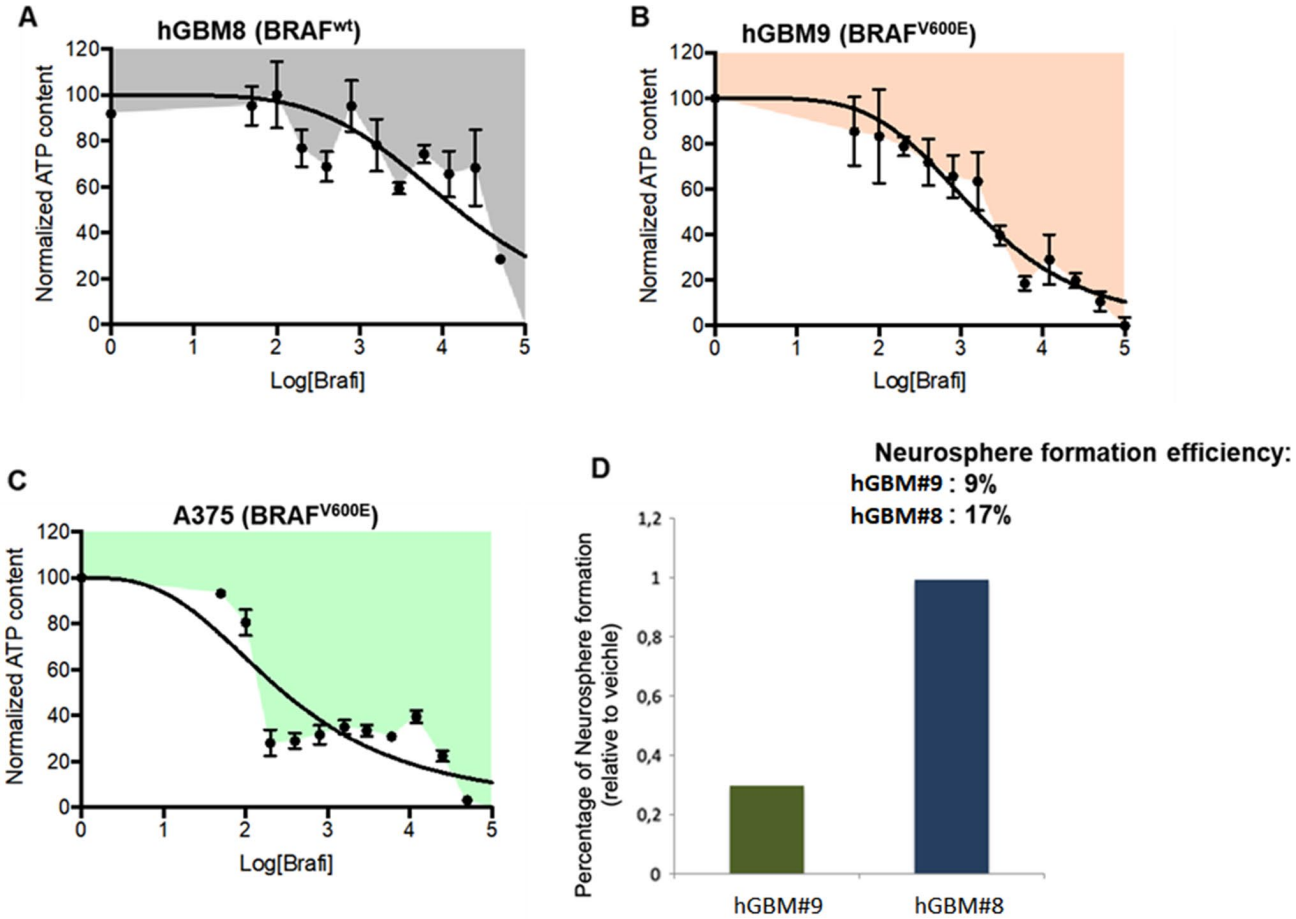
**A**, **B,** BRAF WT hGBM8 and BRAF mutant hGBM9 cells were seeded at 1*10^5^ in 96-well plate in the presence of different concentrations of the BRAF inhibitor Dabrafenib and their viability was determined by the CellTiter-Glo® Luminescent Cell Viability Assay (Promega) after 72 h. Quantitative analyses are shown as curve fitting performed by GraphPad Prism 7.0. Data are means ± SD. **C,** A375 melanoma cells were seeded at 5*10^3^ in 96-well plate in the presence of different concentrations of the same BRAFi for 72 h and then cell viability was assessed as described above. **D**, Effects of Dabrafenib on sphere forming efficiency by hGBM8 and hGBM9 cells

Among all the tested samples, the p.V600E *BRAF* mutation was found in the hGBM#9 GSC sample derived from a recurrent GBM. This mutation was initially confirmed by Sanger sequencing and also by analysis of genomic DNA from the matched parental tumor. That said, this GSC sample has been exploited as a proof-of-concept study for the validation of a drug target. Based on previous studies in other tumor types, the p.V600E mutation was predicted to make tumor cells sensitive to BRAF inhibitors. To investigate this possibility, we treated hGBM9 cells with dabrafenib in vitro. A GSC sample lacking *BRAF* mutation (hGBM8) was also tested as control. The *BRAF*-mutated hGBM9 GSCs were more sensitive to dabrafenib compared with the wild-type *BRAF*-bearing hGBM8 GSCs (IC50 values 5.63 and 50 µM, respectively) (Fig. 3A, B). Interesting, however, the IC50 of dabrafenib in *BRAF*-mutated hGBM8 cells was much higher than in the canonical A375 melanoma cell line (Fig. 3C, IC50 = 0.001 µM) used in previous studies [13]. These results suggest that the genetic and epigenetic make-up of tumor cells arising from different tissues can play a role in shaping the intensity of the drug response.

GSCs are functionally defined as cells sustained by extensive self-renewal ability, which is assessed in vitro by measuring the sphere formation ability in semi-solid medium. Dabrafenib attenuated self-renewal in the *BRAF*-mutated hGBM9 GSCs, while not affecting at all the sphere formation capacity of the wild-type *BRAF* hGBM8 GSCs (Fig. 3D). We conclude that p.V600E *BRAF* mutation confers sensitivity to BRAF inhibitors in vitro, thereby supporting their use for *BRAF*-mutated GBMs.

### Gene expression analysis of GSCs

**Fig. 4:**
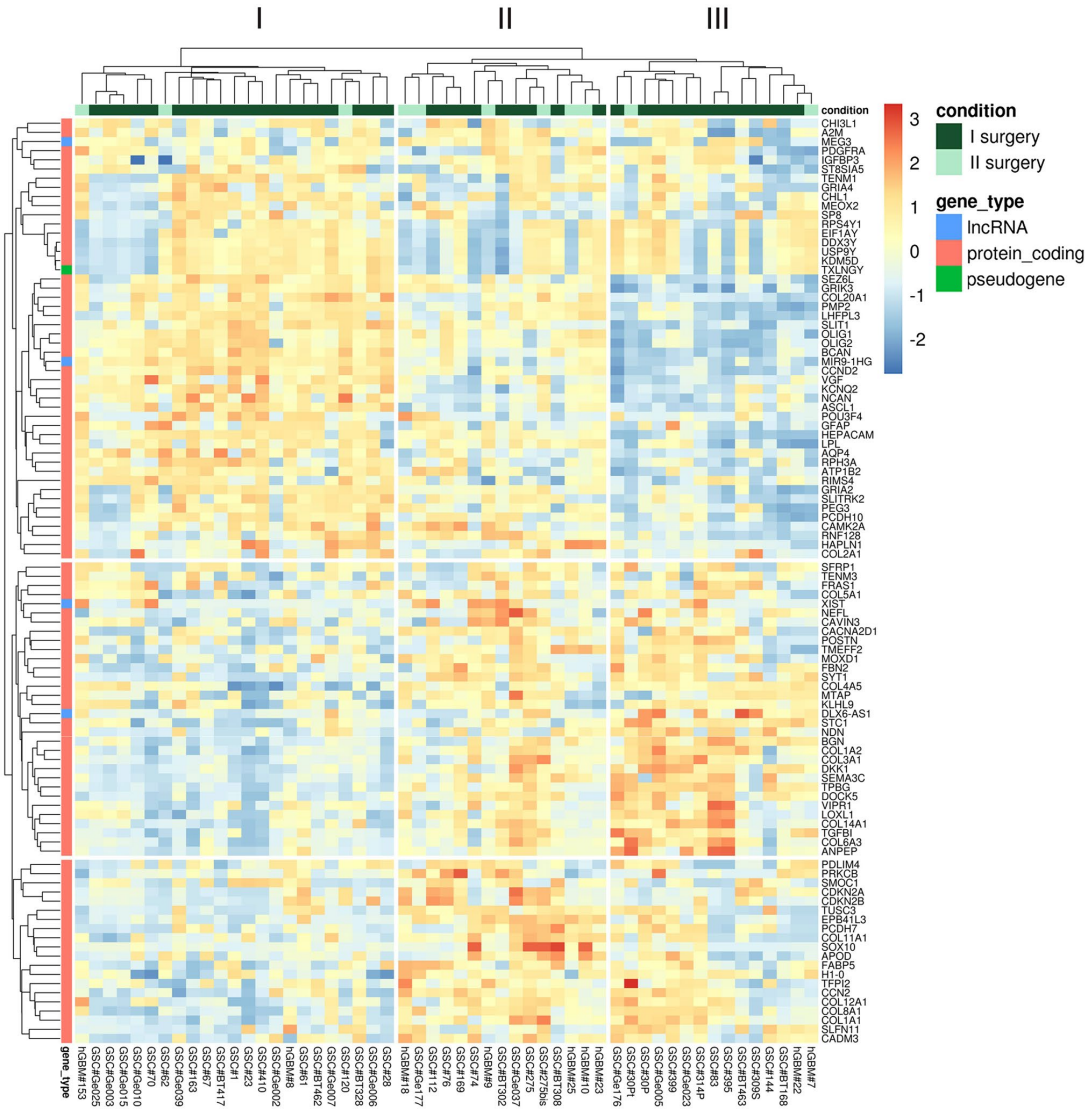
Unbiased clustering of the gene expression profile of 53 lines of GSCs by RNA-seq based on the 100 most variable genes

**Fig. 5:**
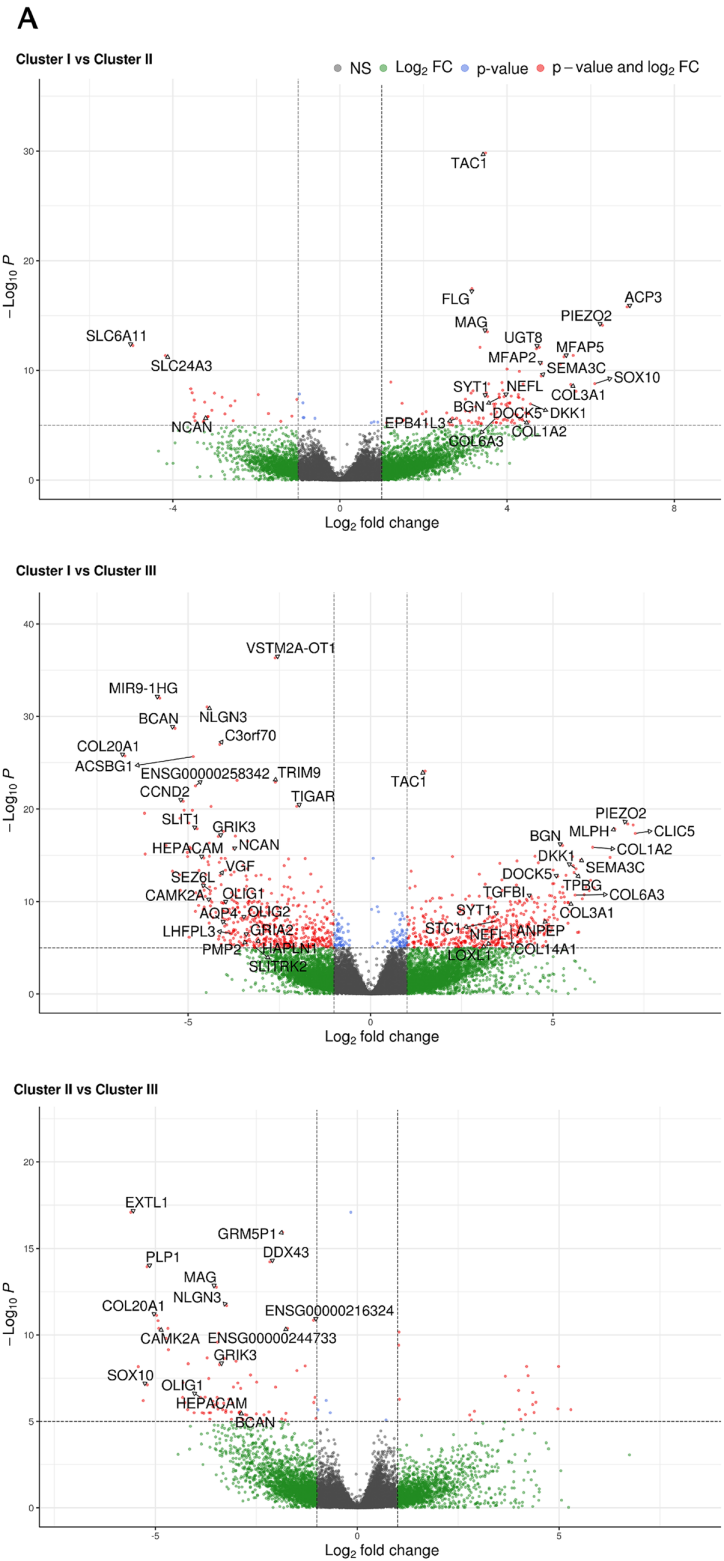

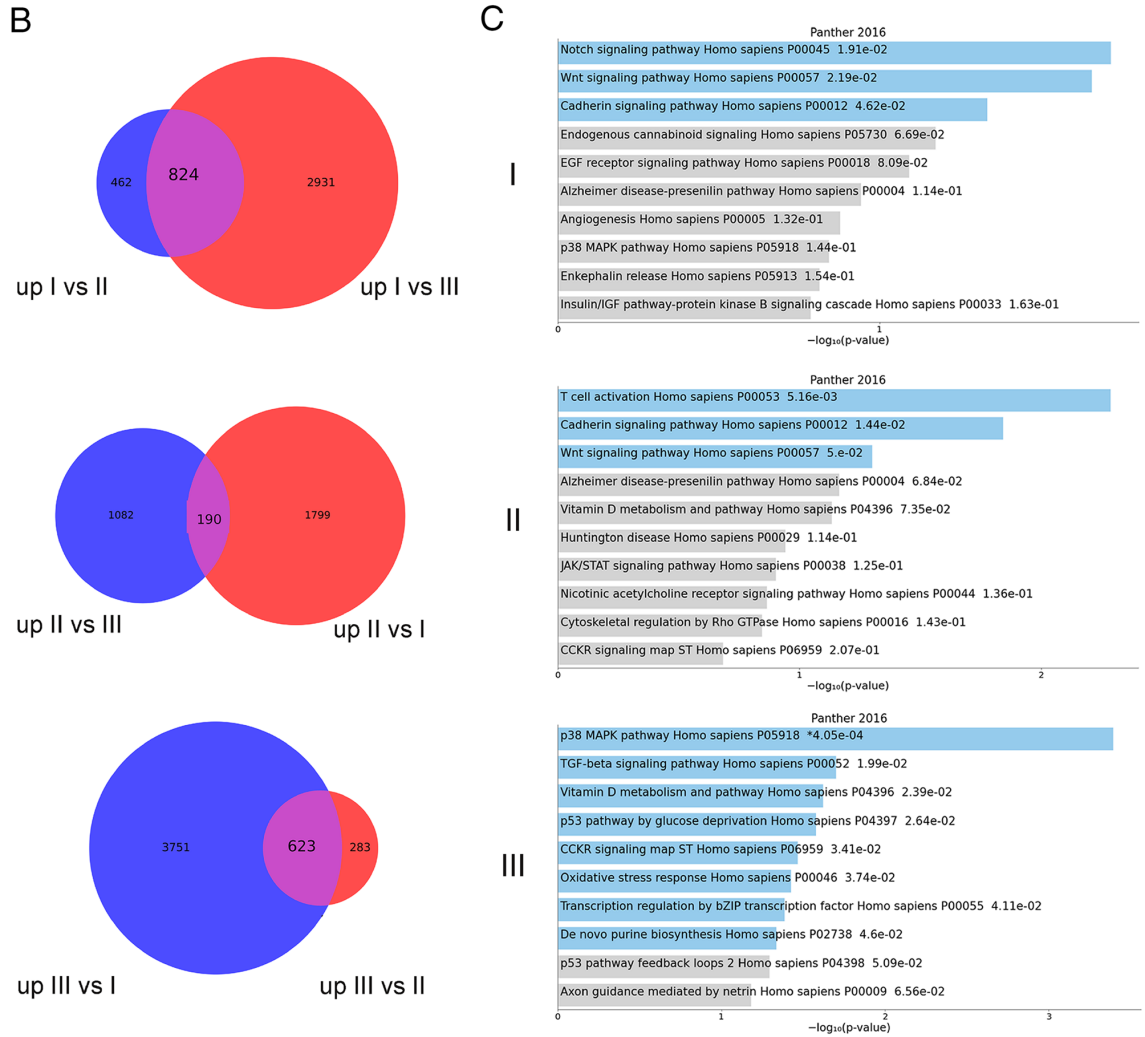
**A,** Volcano plots showing the results of differential expression analyses by comparing the three groups of previously identified samples in pairs. The cut-off for log2FC and for adjusted P value are > 2 and 10e-6. Only the names of the genes present in the previous heatmap or those more up/down-regulated in single pairwise comparisons are reported. **B,** Pathways differentiating the three GSC different clusters identified by gene expression enrichment:Venn diagrams showing the number of specific and common genes in the comparison between each cluster against the other two; the latter were considered as putative “marker genes” for each cluster and gene set enrichment was tested against the Panther (2016) metabolic and cell signaling pathway database. **C,** The bar chart visualizes the top 10 enriched terms and their p-values. Bars are colored based on their p-values, while an asterisk next to a p-value indicates the term also has a significant adjusted p-value (< 0.05). The three different clusters were indicated (I, II and III)

To study in detail a molecular layer of complexity that goes beyond the genetic background and further characterizes GSC lines, we analyzed by deep sequencing the transcriptome from 53 cell lines (42 from primary GBMs, 11 from recurrent GBMs). After alignment, the uniquely mapped reads were used for gene expression quantification. Unsupervised hierarchical clustering based on Euclidean sample distances using the 100 most variable genes produced three distinct GSC clusters (Fig. 4). The grouping of the samples guided by the transcriptome does not reflect the clinical characteristics of the GBM samples which are distributed in a fairly uniform way in each of the three clusters. Differential expression analysis by comparing each GSC cluster with the other two in turn showed, as summarized by the volcano plots (Fig. 5), the greatest differences in terms of significantly up/down-regulated genes (FDR ≤ 0.05) between GSC clusters I and III (with 8129 genes), while the differences were less marked between clusters I and II (3274 genes) or II and III (2178 genes).

Of note *TAC1*, *SYT1*, *COL3A1*, *COL1A2* genes were upregulated in both clusters II and III (Fig. 5A). We observed that in cluster I, *TAC1* gene was not expressed while we found upregulated *OLIG1*, *OLIG2*, *GRIK3/4*, *GRIA2*, and *BEST3*. Among the top genes being overexpressed in cluster II were *SOX10*, *HEPACAM*, *TAC1*, and *MAG*, while cluster III was characterized by the up-regulation of TAC1 and several collagen genes (*COL14A1* and *COL6A3*, *COL3A1*, *COL1A2*) and by the lack of the lncRNA *VSTM2A-OT1* that was present in the other two clusters (Fig. 5A).

In order to identify the “marker” genes of the three GSC clusters, we selected those commonly and significantly up-regulated in each cluster with respect to the other two (Fig. 5B). To gain further insight into the molecular pathways that characterize the three clusters, functional enrichment analysis was performed (based on Panther pathways). Enrichment analysis of the selected genes showed that cluster I was characterized by activation of the Notch and Wnt signalling pathways, followed by the Cadherin pathways (Fig. 5C). Cluster II showed the T-cell immunological activation pathway (Fig. 5C) and cluster III was described by the activation of multiple pathways including p53, p53 by glucose deprivation, p38 MAPK pathways, and TGF-beta signaling pathway (Fig. 5C).

## Discussion

Overall, genomic characterization of the 94 GBM GSC samples unveiled among the top mutated genes several driver genes previously described in brain tumors such as *TP53*, *PTEN*, *RB1, NF1, POLD1, PIK3CA, EGFR*, and *MSH6*, thus indicating that GSCs maintain genetic alterations similar to those found in GBM patients. However, some genes are more frequently mutated in GSCs at diagnosis or relapse. Among the genes more frequently mutated in GSCs at diagnosis we found mismatch repair genes, including *MLH3*, *MSH2*, *MSH4* and *MSH6* (Fig. 3). This finding was also confirmed by KEGG pathway analysis (Fig. 2C), and it is unexpected based on previous studies in patients’ samples reporting higher prevalence of mutations in MMR genes at relapse [1, 14, 15]. However, the fact that our study population is predominantly composed by GSCs derived at diagnosis might lead to the underestimation of these mutations in GSCs obtained at relapse, thus leading to apparent enrichment of MMR gene mutations in GSCs established at diagnosis.

Intriguingly, among genes more mutated in GSCs at relapse (Fig. 2), we found *ACAD9* and *ACAD10*, two members of the Acetyl-Carboxylase Dehydrogenase family which contributes to β-oxidation of fatty acids (FA) in mitochondria [16]. These mutations might underscore different metabolic features of GBM at relapse as compared with those retrieved at diagnosis, which will deserve investigation in future studies.

With regard to additional, specific genetic traits enriched in GCSs obtained at relapse, KEGG pathway analysis uncovered increased MAPK signaling as well as FGFR and KIT signaling (Fig. 4). Of note, the multi-kinase inhibitor regorafenib, which also interferes with FGFR, KIT and MAPK signaling [17], has shown promising therapeutic activity in relapsed GBM [18]. These genetic data should stimulate functional studies comparing therapeutic activity of specific MAPK inhibitors on GSCs from diagnosis versus relapse in order to strengthen the hypothesis that MAPK inhibitors could be more active on relapsed GBM.

We validated the general concept that activating mutations in driver genes correlate with sensitivity to specific inhibitors, with the *BRAF* V600E mutant hGBM8 GSC line, which was shown to be 10-fold more sensitive to a BRAF inhibitor in vitro compared with *BRAF* WT hGBM9 cells (Fig. 3). A clear-cut effect of treatment on sphere formation capability of GSC cells was also detected, supporting the therapeutic activity of BRAF inhibitors of patients with *BRAF* mutant GBMs [19].

Unsupervised hierarchical clustering of deep sequencing analysis data generated from 53 patient-derived GSCs transcriptomes (established from 42 primary GBMs and 11 recurrent GBMs, respectively), identified three distinct GSC clusters. Enrichment analysis of the genes characterizing the three clusters (Panther), revealed that cluster I and cluster II shared activation of common pathways such as Wnt and Cadherin. Conversely, cluster III was characterized by the activation, among others, of TGF-beta signaling pathway.

In GBM, alteration and/or upregulation of the Wnt and/or TGF-beta pathway is associated with pathogenesis of the disease and aggressive tumor behavior [20]. The increased activity of the canonical Wnt pathway may be responsible for the resistance to chemotherapy and radiotherapy, as well as growth, aggressiveness and invasive potential of GBM [20]. Moreover, β-catenin is the unique marker of proliferating endothelial cells in GBM [21], considered another feature of aggressiveness.

Lymphoid enhancer-binding factor 1 (LEF1), a member of the T-cell Factor (TCF)/LEF1 family of high-mobility group transcription factors, is a downstream mediator of the Wnt/β-catenin signaling pathway LEF1 is essential in stem cell maintenance and organ development, especially in its role in epithelial-mesenchymal transition (EMT) by activating the transcription of hallmark EMT effectors including N-Cadherin, Vimentin, and Snail, resulting in cancer progression [22]. The activation of T-cell activation signaling pathway in cluster II reinforces the role of LEF1 in modulating gene transcription also independently from the activation of Wnt/β-catenin signaling pathway promoting GBM cells invasion, migration, proliferation, and the self-renewal potential of GSCs [23].

Additionally, the activation of Notch signaling pathways in cluster I may contribute to intratumor heterogeneity by promoting stem cell behavior in GSCs and regulates multiple steps of gliomagenesis, including tumor initiation, progression and recurrence [24].

Cluster III is characterized by activation of TGF-beta signaling and p38 MAPK pathways. TGFβ mediates inflammatory signaling pathways in normal tissues whereas, in tumors it alters the cell cycle and mediates malignant features [25] and immune suppression [26], specifically in GBM [27]. Previous studies have demonstrated that TGF-β activity is present in aggressive and highly proliferative glioma [28]. TGF-β has been shown to induce self-renewal capacity and prevent differentiation in GSCs. Furthermore, TGF-β may play a role in GSC-mediated oncogenesis via leukemia inhibitory factor induction in vivo [29]. Activation of p38 MAPK could contribute to maintain GSCs in an undifferentiated state [30].

Among the 100 most variable genes contributing the GCSs clustering into three groups, we found three long non coding RNAs (lncRNAs): MEG3, XIST and DLX6-AS1. Recent studies have highlighted the potential roles of these transcripts as diagnostic and prognostic biomarkers in several types of human cancers, including GBMs. Expression levels of lncRNAs can identify GBM patients from healthy subjects, and they can potentially distinguish specific brain tumors [31]. Moreover, growing evidences suggest that lncRNAs can be valuable as potential targets for anticancer therapy. MEG3 plays a pivotal role in GBM proliferation, migration, EMT and notably, has been associated with poor overall survival [32]. MEG 3 exerts its role in GBM pathology by interacting with tumor suppressor regulatory miRNAs [33]. Noteworthy, abnormal activation of Wnt pathway induces assembly of β-catenin in the nucleus stimulating transcription of several oncogenes, while high expression of MEG3 could suppress cell proliferation by inactivating Wnt/β-catenin signaling pathway [34]. The oncogenic lncRNAs, XIST plays a major role in regulating cell cycle leading to increased tumor growth and invasion. The underlying mechanisms include Bcl-2 expression regulation, upregulation of CREB1 and regulation of IRS1/PI3K/Akt pathway via cross-talk with other ncRNAs [35, 36].

High expression and association with poor prognosis in GBM patients have been reported also for the other oncogenic lncRNAs, DLX6-AS1. This lncRNA has been shown to promote GBM cell growth, proliferation, invasion by modulation of the DLX6-AS1/miR-197-5p/E2F1 axis being both miR-197-5p and the transcription factor E2F1, target genes of the lncRNA in GBM [37].

Taken together, the genomic and transcriptomic data on GSCs reported in this multicentric study allowed the generation of novel insights in alternative therapeutic approaches and both significantly contributed to provide a novel and detailed and integrated picture of the potential diagnostic and prognostic biomarkers in GBM.

## Supplementary Information

Below is the link to the electronic supplementary material.

Supplementary material 1 (DOCX 748.4 kb)

## Data Availability

The datasets generated during the current study are available in the NCBI Sequence Read Archive database under accession number SUB12144948 (https://www.ncbi.nlm.nih.gov/sra).
