# Supplementary material for: Genome-wide profiling of patient-derived glioblastoma stem-like cells reveals recurrent genetic and transcriptomic signatures associated with brain tumors
Source: J Neurooncol. 2023 May 4;163(1):47–59. doi: 10.1007/s11060-023-04287-6 (PMC10232615; doi:10.1007/s11060-023-04287-6)
Supplement: Supplementary file 1 — Supplementary material 1 (DOCX 748.4 kb) [file 11060_2023_4287_MOESM1_ESM.docx]

**SUPPLEMENTARY INFORMATION**

**Supplementary Methods**

**Whole exome sequencing (WES) analyses**

Read quality was assessed by FastQC and Trimmomatic was used to select high-quality reads (phred score >20) and eliminate adapter sequences. The high-quality reads were mapped against reference genome hg38 using BWA. Preprocessing and refinement of alignment files for variant calling was done in accordance with GATK best practices available at <https://software.broadinstitute.org/gatk/best-practices/>. mutect2 was used was used to call variants. To eliminate potential sequencing and germline artifacts and other sources of false-positive calls that may be present in the VCF files, a series of filtering steps were performed. Calls that were marked as germline SNPs in at least two samples in a “Panel of Normals” (PoN) dataset were filtered out. The PoN dataset was built from SNPs/germline variants called by the GATK’s HaplotypeCaller across i) our 12 normal blood samples and ii) the public available panels of normal from the gnomAD [[1](#_ENREF_16)] and the 1000 genomes [2] project. Identified variants were filtered for depth of >10 and QUAL >20 and annotated with SnpEff on clinvar, cosmic and dbsnp databases. Finally, we filtered out all the variants annotated as LOW and MODERATE impact (maintaining only those on genes known to be involved in GBM) and benign effect. The list of the genes of the selected variants was then processed to find enriched functional categories. Enrichment analyses were performed using clusterprofiler R package on Gene Ontology (GO) categories as well as Reactome and KEGG pathway databases.

**RNA-Seq analysis methods**

In the preprocessing step the raw reads in fastq format were inspected and cleaned using FASTP [3]. The mean quality per base was fixed at a phred-score of 20 and reads with more than 30% of unqualified bases (-q 20 -u 30 -l 55 --detect_adapter_for_pe) were removed. Reads shorter than 55 bases were also removed.

Cleaned reads were aligned with STAR (2.7.9a) [4] using the ENCODE standard options onto a consensus version of the reference (GRCh38) human genome. The 1000 Genomes Project VCF file with consensus SNVs and InDels was provided at the genome generation stage and the alternative alleles in this VCF have been inserted to the reference genome to create a ”transformed” genome. At the mapping stage, the reads have been mapped to the transformed genome and the alignments were transformed back to the original (reference) coordinates.

Gene expression was quantified with featureCounts [5] using the Gencode (release 39) reference gene annotation taking advantage of the strand-oriented nature of the reads. The row counts were normalized with DESeq2 [6] and a batch effect correction was introduced with the R limma package [7] taking into account the origin of glioma cell lines from different institutes. Vsd-modified normalized counts were used to select the top 100 genes ranked by row variance and unsupervised hierarchical clustering between samples was performed on the basis of these genes. The resulting heatmap was generated with the R *pheatmap* library highlighting also clusters of co-regulated genes. Inter-cluster differential gene expression analysis was conducted with DESeq2. The volcano plots were generated with the R *EnhancedVolcano* library. The Venn diagrams were generated with the specific matplotlib-venn python library and the gene set enrichment was tested with the EnrichR (https://maayanlab.cloud/Enrichr/) web tool.

**SUPPLEMENTARY REFERENCES**

1. Karczewski KJ, Francioli LC, Tiao G et al. The mutational constraint spectrum quantified from variation in 141,456 humans. Nature 2020; 581: 434-443.

2. Fairley S, Lowy-Gallego E, Perry E, Flicek P. The International Genome Sample Resource (IGSR) collection of open human genomic variation resources. Nucleic Acids Res 2020; 48: D941-D947.

3. Chen S, Zhou Y, Chen Y, Gu J. fastp: an ultra-fast all-in-one FASTQ preprocessor. Bioinformatics 2018; 34: i884-i890.

4. Dobin A, Davis CA, Schlesinger F et al. STAR: ultrafast universal RNA-seq aligner. Bioinformatics 2013; 29: 15-21.

5. Liao Y, Smyth GK, Shi W. featureCounts: an efficient general purpose program for assigning sequence reads to genomic features. Bioinformatics 2014; 30: 923-930.

6. Love MI, Huber W, Anders S. Moderated estimation of fold change and dispersion for RNA-seq data with DESeq2. Genome Biol 2014; 15: 550.

7. Ritchie ME, Phipson B, Wu D et al. limma powers differential expression analyses for RNA-sequencing and microarray studies. Nucleic Acids Res 2015; 43: e47.

**SUPPLEMENTARY FIGURES LEGENDS.**

**Supplementary Figure 1. (A)** GO analysis using the complete list of mutated genes in the GSCs. **(B-C)** GO analysis of molecular function (panel B) and cellular component analysis (panel C) **(D)** Main mutational signatures in GSCs according to Reactome database analysis. **(E)** Main mutational signatures in GSCs according to KEGG database analysis.

**Supplementary Figure 2.** TreePlots representing the Gene Ontology hierarchy of the significantly (FDR<0.05) enriched (A) biological process, (B) molecular function and (C) cellular component categories.

**Supplementary Table 1**. GBM cell lines: clinical features.

**Supplementary Figure 1.**

**
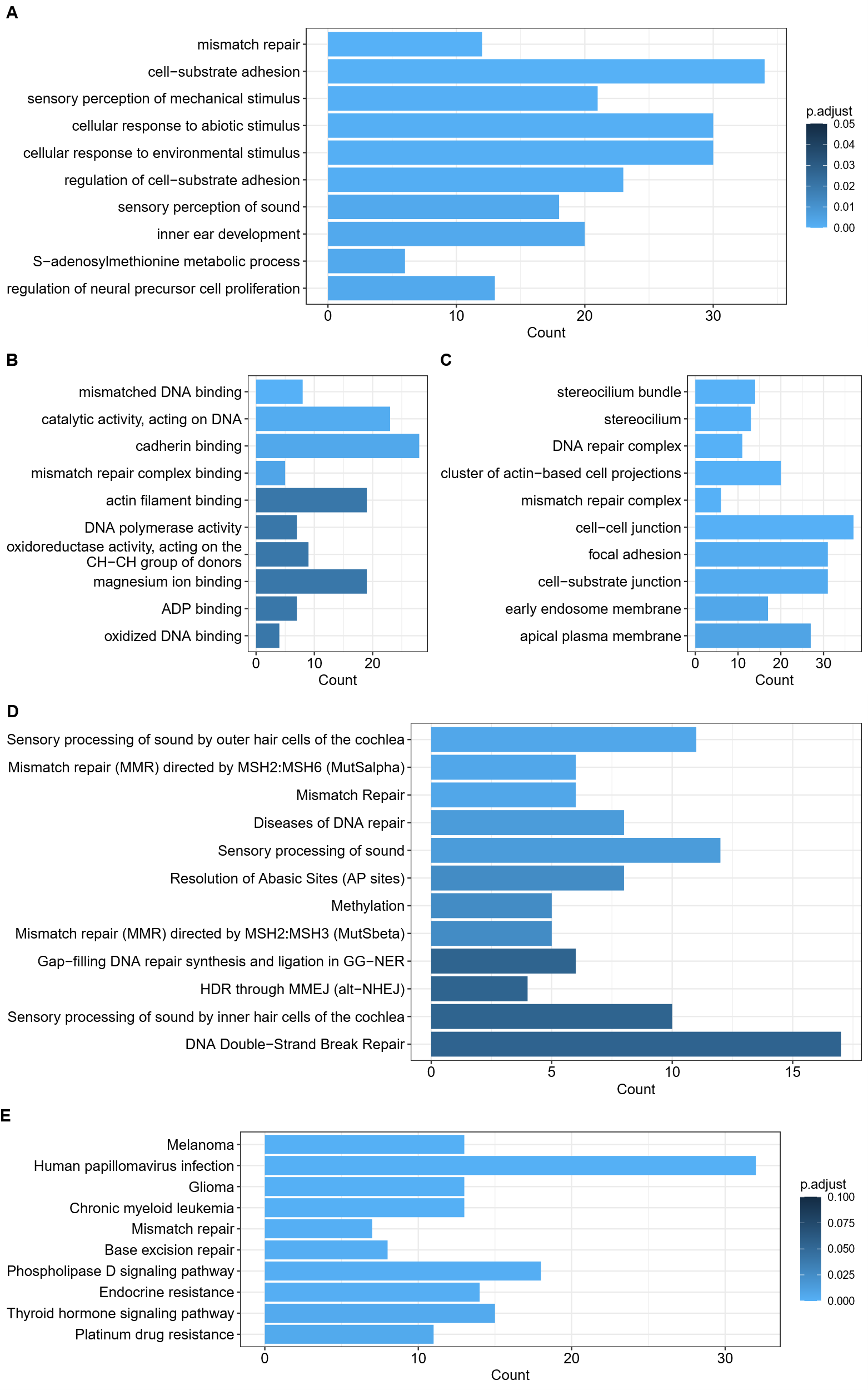
**

**Supplementary Figure 2.**

**Supplementary table 1.**

| Cell line ID | Age/Sex | Tumor location | Type of tumor | Ki67 (%) | *MGMTp* | *IDH*1/2 | OS (mos) |
| --- | --- | --- | --- | --- | --- | --- | --- |
| **GSC#1** | 40/M | temporal | P | 20 | M | wt | 12.5 |
| **GSC#23** | 77/M | parietal | P | 50 | UM | wt | 2.0 |
| **GSC#28** | 72/M | frontal | P | 5 | M | wt | 11.5 |
| **GSC#30P** | 44/M | frontal | P | 10 | M | wt | 7.5 |
| **GSC#30Pt** | 44/M | frontal | P | 10 | M | wt | 7.5 |
| **GSC#61** | 59/M | occipital | P | 35 | UM | wt | 6.0 |
| **GSC#62** | 64/M | frontal | R | 10 | M | wt | 14.0 |
| **GSC#67** | 48/M | parietal | P | 20 | UM | wt | 2.0 |
| **GSC#70** | 67/F | parietal | P | 20 | UM | wt | 9.0 |
| **GSC#74** | 70/F | frontal | P | 15 | UM | wt | 8.0 |
| **GSC#76** | 48/F | frontal | P | 15 | UM | wt | 16.0 |
| **GSC#83** | 52/M | temporal | P | 40 | UM | wt | 8.0 |
| **GSC#112** | 49/F | parietal | P | 18 | M | wt | 6.0 |
| **GSC#120** | 53/M | parietal | R | 30 | UM | wt | 16.5 |
| **GSC#144** | 57/M | temporal | P | 40 | M | wt | 19.0 |
| **GSC#147** | 69/F | frontal | P | 25 | UM | wt | 11.0 |
| **GSC#151** | 69/M | occipital | P | 30 | M | wt | 72.0 |
| **GSC#163** | 56/M | parietal | P | 15 | UM | wt | 2.0 |
| **GSC#169** | 61/M | temporal | P | 40 | UM | wt | 9.0 |
| **GSC#184** | 53/F | frontal | P | 25 | UM | wt | 15.0 |
| **GSC#208** | 68/M | temporal | R | 40 | UM | wt | 33.0 |
| **GSC#210** | 53/M | parietal | P | 40 | UM | wt | 10.5 |
| **GSC#213** | 49/M | frontal | R | 30 | M | wt | 27.0 |
| **GSC#220C** | 62/M | frontal | P | 30 | UM | wt | 2.0 |
| **GSC#257** | 57/M | multicentric | P | 50 | M | wt | 23.0 |
| **GSC#275** | 58/M | occipital | P | 50 | M | wt | 12.0 |
| **GSC#275bis** | 59/M | occipital | R | 50 | M | wt | 12.0 |
| **GSC#284** | 60/M | frontal | P | 40 | UM | wt | 3.0 |
| **GSC#309S** | 65/M | temporal | P | 5 | M | wt | 13.5 |
| **GSC#314P** | 54/F | parietal | P | 40 | M | wt | 8.0 |
| **GSC#393** | 70/M | temporal | P | 25 | UM | wt | 8.5 |
| **GSC#395** | 49/M | temporal | P | 25 | M | wt | 7.5 |
| **GSC#399** | 67/M | temporal | P | 20 | M | wt | 15.5 |
| **GSC#410** | 67/M | frontal | P | 25 | UM | wt | 13.5 |
| **GSC#Ge002** | 53/M | parietal | P | 20 | UM | n.a. | 17.0 |
| **GSC#Ge003** | 49/M | occipital | P | 40 | UM | n.a. | 14.4 |
| **GSC#Ge005** | 67/M | occipital | P | 40 | M | n.a. | 9.5 |
| **GSC#Ge006** | 51/M | temporal | P | 40 | UM | n.a. | n.a. |
| **GSC#Ge007** | 71/M | frontal | P | 50 | n.a. | n.a. | 3.6 |
| **GSC#Ge010** | 70/F | frontal | P | 40 | M | n.a. | 37.9 |
| **GSC#Ge015** | 58/M | parietal | P | 20 | M | n.a. | 17.9 |
| **GSC#Ge023** | 70/F | parietal | P | n.a. | M | n.a. | 7.3 |
| **GSC#Ge025** | 81/M | temporal | P | 25 | M | n.a. | 14.3 |
| **GSC#Ge037** | 73/M | parietal | P | 30 | n.a. | n.a. | 9.9 |
| **GSC#Ge039** | 47/M | occipital | P | 25 | n.a. | n.a. | 10.4 |
| **GSC#Ge176** | 57/F | n.a. | P | n.a. | n.a. | n.a. | 18.3 |
| **GSC#Ge177** | 68/F | n.a. | P | n.a. | n.a. | n.a. | 14.2 |
| **L160315** | 68/F | temporal | P | n.a. | n.a. | n.a. | 12.2 |
| **L160525** | 73/F | temporal | P | 35 | n.a. | wt | 15.7 |
| **L160526** | 59/M | FTP | P | 40 | n.a. | wt | 10.2 |
| **L160622** | 60/M | temporal | P | 30 | n.a. | wt | n.a. |
| **L160704** | 47/M | frontal | P | 60 | n.a. | wt | 12.1 |
| **L161019** | 47/M | frontal | P | 20 | n.a. | wt | 8.7 |
| **L161205** | 56/M | frontal | P | 25 | n.a. | wt | 47.2 (alive) |
| **L1312** | 59/F | FTP | P | 35 | n.a. | n.a. | n.a. |
| **L0627** | n.a. | n.a. | n.a. | n.a. | n.a. | n.a. | n.a. |
| **L0605** | n.a. | n.a. | n.a. | n.a. | n.a. | n.a. | n.a. |
| **L0104** | n.a. | n.a. | n.a. | n.a. | n.a. | n.a. | n.a. |
| **L160503** | 70/M | frontal | P | 20 | n.a. | wt | 9.4 |
| **G40** | 81/M | Temporal | P | 15 | UM | *IDH1* wt | 2 |
| **G41** | 69/M | Temporal | P | 15 | M | *IDH1* wt | 45 |
| **G48** | 76/M | Temporal | P | 30 | M | n.a. | 10 |
| **G63** | 70/F | Temporal | P | 30 | M | n.a. | 7 |
| **G65** | 58/M | Frontal | P | 25 | M | *IDH1* wt | 22 |
| **G70** | 48/M | Frontal | P | 15 | M | *IDH1* wt | 30 |
| **G74** | 72/M | Frontal | P | 15 | M | *IDH1* wt | 17 |
| **G80** | 40/M | Temporal | P | 50 | M | *IDH1* wt | 57 * |
| **hGBM#8** | 41/M | n.a. | P | 35 | M | wt | n.a. |
| **hGBM#22** | 77/M | n.a. | P | 50 | UM | wt | 9,0 |
| **hGBM#9** | n.a./F | temporal | R | 5 | UM | wt | 55,0 |
| **hGBM#18** | 26/M | parietal | R | 40 | M | wt | 14.0 |
| **hGBM#10** | n.a./M | n.a. | R | 40 | M | wt | 55.0 |
| **hGBM#161** | 69/M | frontal | P | n.a. | M | wt | 12.0 |
| **hGBM#23** | 58/M | temporal | P | 25 | M | wt | 14.0 |
| **hGBM#7** | 54/M | n.a. | R | 50 | UM | wt | n.a. |
| **hGBM#20** | 63/F | parietal | P | 40 | n.a. | n.a. | 15.0 |
| **hGBM#153** | 66/M | n.a. | P | n.a. | n.a. | n.a. | n.a. |
| **GSC#BT155** | 45.6/F | temporal | P | n.a. | UM | wt | 13 |
| **GSC#BT168** | 77,3/F | parietal | P | n.a. | UM | wt | 6 |
| **GSC#BT274** | 48.5/M | temporal | P | n.a. | n.a. | n.a. | 18 |
| **GSC#BT302** | 18/F | rolandic | P | n.a. | UM | wt | 146 (alive) |
| **GSC#BT308** | 76.3/M | temporal | P | n.a. | UM | wt | 5 |
| **GSC#BT328** | 54.4/M | rolandic | P | n.a. | UM | wt | 18 |
| **GSC#BT330** | 58.7/M | frontal | P | n.a. | n.a. | n.a. | 35 |
| **GSC#BT332** | 74.2/M | occipital | P | n.a. | UM | wt | 9 |
| **GSC#BT337** | 63.7/M | temporal | P | n.a. | UM | wt | 17 |
| **GSC#BT404** | 57.8/M | frontal | P | n.a. | UM | wt | 9 |
| **GSC#BT417** | 50.7/M | frontal | P | n.a. | M | wt | 23 |
| **GSC#BT462** | 70.8/M | frontal | P | n.a. | UM | wt | 6 |
| **GSC#BT463** | 69.8/M | temporal | P | n.a. | UM | wt | 11 |
| **GSC#BT483** | 53.5/M | temporal | P | n.a. | M | wt | 8 |
| **GSC#BT296** | 69.1/F | frontal | P | n.a. | n.a. | n.a. | 3 |
| **GSC#BT306** | 54.9/M | temporal | R | n.a. | n.a. | n.a. | 2 |
| **GSC#BT318** | 69.7/M | occipital | P | n.a. | n.a. | n.a. | 17 |
| **hGBM#25** | n.a. | n.a. | n.a. | n.a. | n.a. | n.a. | n.a. |

Legend. P., Primary tumor; R., Recurrent tumor; M., methylated; UM, unmethylated; wt, wild type; mut, mutated; OS, overall survival; n.a., not available.
